# Subconductance states in NMDA receptor variants are Ca^2+^ impermeable and act at the M2 loop

**DOI:** 10.21203/rs.3.rs-10155714/v1

**Published:** 2026-07-07

**Authors:** Bohdan Kysilov, Ramesh Prasad, Erica Nebet, Joseph Bennett, Johansen B. Amin, Huan-Xiang Zhou, Lonnie P. Wollmuth

**Affiliations:** 1 Graduate Program in Neuroscience, Stony Brook University, Stony Brook, NY 11794-5230; 2 Graduate Program in Molecular and Cellular Pharmacology, Stony Brook University, Stony Brook, NY 11794-5230; 3 Medical Scientist Training Program (MSTP), Stony Brook University, Stony Brook, NY 11794-5230; 5 Department of Chemistry, University of Illinois at Chicago, Chicago, IL 60607; 6 Department of Physics, University of Illinois at Chicago, Chicago, IL 60607; 7 Center for Nervous System Disorders, Stony Brook University, Stony Brook, NY 11794-5230; 8 Department of Neurobiology & Behavior, Stony Brook University, Stony Brook, NY 11794-5230; 9 Department of Biochemistry & Cell Biology, Stony Brook University, Stony Brook, NY 11794-5230

**Keywords:** Synaptic transmission, ion channels, ionotropic glutamate receptors, *de novo* missense mutations, single channel recordings, Ca^2+^ permeability

## Abstract

A variety of *de novo* disease-associated variants are found in NMDA receptors (NMDARs), which carry out critical roles at synapses including mediating a Ca^2+^ influx. In the central permeation pathway, a vestibule is positioned between the external M3 gate and the M2 pore loop. A variant in the obligatory GluN1 subunit, Met641Ile, reduces Ca^2+^ permeability, but the mechanism of action is unknown. We find that other variants at GluN1-M641, including Leu, Val, and Thr, as well as variants at a homologous position in GluN2A (Val639Ile), do not alter Ca^2+^ permeability. Notably, and in contrast to the other variants, GluN1-Met641Ile has a prominent subconductance state. Molecular dynamics simulations show that the Ile641 side chain interacts more with the M2 loop, including the N-site in GluN1 and GluN2, than the wild-type Met641 side chain. N-site variants also induce subconductance states and reduce Ca^2+^ permeability. We conclude that subconductance states are largely Ca^2+^ impermeable and that the Met641Ile variant alters Ca^2+^ permeability by inducing a subconductance state via interacting with the M2 loop.

## INTRODUCTION

NMDA receptors (NMDARs) are ligand-gated ion channels that participate in brain development^1–3^ and mediate excitatory synaptic transmission throughout the central nervous system^4,5^. NMDARs also contribute to acute and chronic neurologic and psychiatric disorders when their activity is aberrant^6–8^. Of note, many *de novo* missense mutations in the *GRIN* genes encoding NMDAR are associated with neurodevelopmental disorders, including seizures, intellectual disability, movement disorders, and schizophrenia^9–11^.

NMDARs possess unique ion channel properties, including high permeability to Ca^2+^, voltage-dependent channel block by Mg^2+^, and slow deactivation rates, which allow them to regulate Ca^2+^ influx in a dynamic manner^2,4,12^. The magnitude of NMDAR-mediated Ca^2+^ influx is fundamental to neuronal maturation, synaptic development, and synaptic plasticity. In terms of plasticity, for example, low, sustained levels of NMDAR activation lead to Ca^2+^-dependent signaling cascades in synaptic depression^13^. In contrast, intense transient NMDAR activation leads to higher levels of Ca^2+^ and synaptic potentiation. Finally, excessive NMDAR-mediated Ca^2+^ influx is associated with cell death in a process termed excitotoxicity^14^. Hence, variations in NMDAR-mediated Ca^2+^ influx are fundamental to their role in nervous system function and pathology.

In addition to Ca^2+^, the main ionic species in the NMDAR-mediated influx is Na^+^. Through a variety of pathways, missense variants can lead to either a gain-of-function (GoF) or a loss-of-function (LoF) in excitatory synaptic transmission. Importantly, changes in intrinsic Ca^2+^ permeability can alter the relationship between the magnitude of Na^+^-dependent excitation and Ca^2+^ influx^15^, complicating efforts to relate GoF or LoF to clinical phenotypes. Hence, defining the impact of variants on intrinsic Ca^2+^ permeability and understanding the mechanisms of how variants alter Ca^2+^ permeability is critical to defining disease pathology.

**Figure 1.**
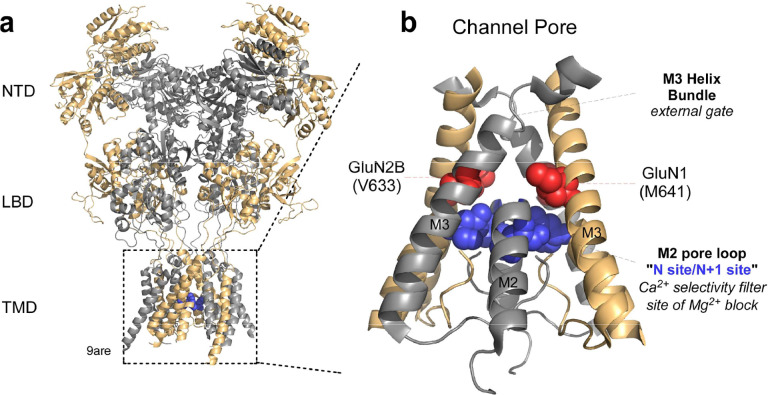
Topology of NMDARs and disease-associated variants in the vestibule. **(a)** Structure of the agonist-bound rat GluN1/GluN2B receptor in the ‘open’ conformation (9ARE (Chou, 2024)). GluN1 and GluN2B subunits are shown in orange and gray, respectively. iGluRs are composed of four highly modular domains: the extracellular N-terminal (**NTD**) and ligand-binding (**LBD**) domains; the membrane-spanning transmembrane domain (**TMD**) that forms the ion channel; and the intracellular C-terminal domain (**CTD**), which is not included in the structure. **(b)** Enlarged view of the TMD elements lining the ion channel pore: The extracellular end is lined by the M3 transmembrane segments, whereas the intracellular half by the non-membrane spanning M2 pore loop. The M3 segments, predominantly the highly conserved SYTANLAAF motif, form an activation gate at the bundle crossing. Asparagine residues GluN1(N616), GluN2B(N615), and GluN2B(N616), which are involved in forming Ca^2+^ selectivity filter and Mg^2+^ binding site, are highlighted in blue. A position in the vestibule with disease-associated variants, GluN1(M641), and its homologous position in GluN2B (V640), are highlighted in red.

NMDARs are highly modular proteins composed of four distinct domains (Figure 1a)^16–19^. The core gating machinery is found in the ligand binding domain (LBD) and the transmembrane domain (TMD), where agonist binding, typically glutamate and glycine, in the LBD translates energy to the TMD, initiating channel opening^20–26^. It is in this core gating machinery that most variants causing the most severe disease phenotypes appear^27–30^.

The TMD forms the ion channel, which in the open conformation forms the permeation pathway where ions can cross the membrane^25,31,32^. The ion channel pore on the extracellular side is formed by the M3 transmembrane helix, where an external gate resides, and on the intracellular side by the M2 reentrant pore loop, which controls Ca^2+^ permeability and Mg^2+^ block^2^ and contains an internal gate^33^ (Figure 1b). Within the M3 segments resides the SYTANLAAF motif, the most conserved motif in this receptor family^34^. The SYTANLAAF motif contain residues that form the external gate^31,32^ and other critical structural elements involved in receptor gating^25,35–37^. Numerous variants are located within this region^30^. Similarly, a variety of variants are found within the M2 pore loop^38,39^.

Positioned between the SYTANLAAF motif and the tip of the M2 loop is a vestibule lined by the M3 segments (Figure 1b). Variants in this vestibule are associated with clinical phenotypes (Supplementary Table 1). They can alter receptor gating and Ca^2+^ permeability^15,40,41^, but the underlying mechanisms are unknown. To further define the role of the vestibule in ion channel function, we characterized variants at GluN1-M641 and homologous positions in the GluN2A (V639) and GluN2D (V667) subunits (highlighted in red in Figure 1b). Variants at GluN1-M641 either had no effect on single channel open probability or significantly reduced it, whereas in contrast those in the GluN2 subunit strongly enhanced receptor gating. Notably, one variant, GluN1(M641I), which was the only variant to reduce Ca^2+^ permeability also strongly enhanced subconductance states. Molecular dynamics simulations revealed increased interactions between the isoleucine replacement at GluN1-M641 and the GluN1 and GluN2 M2 loop, including the N-site asparagines. Variants at the GluN1 and GluN2A N-sites also induce subconductance states and reduce Ca^2+^ permeability. We propose that subconductance states are largely Ca^2+^ impermeable and that the M2 pore loop is a major site regulating conductance levels in NMDARs.

## RESULTS

NMDARs are obligate heterotetramers composed of two GluN1, encoded by *GRIN1*, and typically two GluN2 (A-D), encoded by *GRIN2A-D*, subunits^2^.

### In the vestibule, only the missense variant GluN1a-M641I alters Ca^2+^ permeability

**Figure 2.**
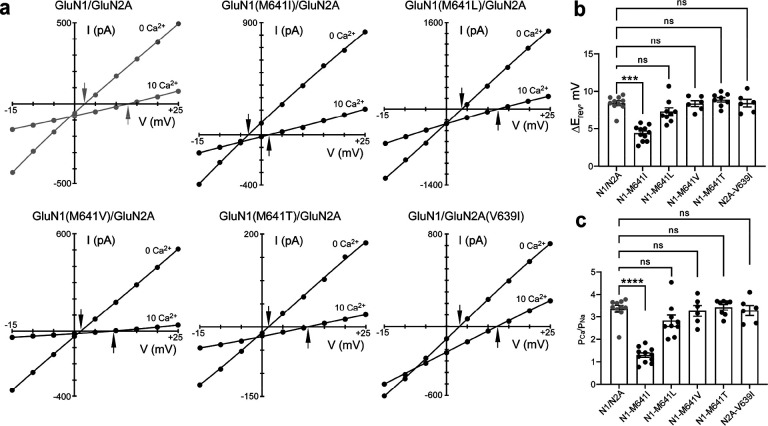
Of tested vestibule disease-associated variants, only GluN1-M641I reduces Ca^2+^ permeability. **(a)** NMDAR current-voltage (IV) relationships for wild-type and disease variants GluN1(M641I)/GluN2A, GluN1(M641L)/GluN2A, GluN1(M641V)/GluN2A, GluN1(M641T)/GluN2A, and GluN1/GluN2A(V639I) expressed in HEK293T cells in an external solution containing 140 mM Na^2+^ and either 0 or 10 mM added Ca^2+^. Arrows indicate reversal potentials (E_rev_). Currents were elicited by 1 mM glutamate in the continuous presence of 100 μM glycine. The 0 Ca^2+^ IV is the average recorded before and after the 10 mM Ca^2+^ recording. **(b & c)** Bar graphs (mean ± SEM) showing changes in E_rev_ (ΔE_rev_) (**b**) and relative Ca^2+^ permeability (P_Ca_/P_Na_) (**c**) of human GluN1/GluN2A (N1/N2A) and tested vestibule variants. P_Ca_/P_Na_ was derived from ΔE_rev_ going from 0 to 10 mM Ca^2+^ using the Lewis equation (see Methods). Number of recordings, from left to right: 10, 11, 9, 6, 8, 6. In this and all subsequent figures, filled circles represent individual data points for wild-type (gray) or variants (black). Statistical comparisons were performed using one-way ANOVA with post hoc Dunnett tests versus wild type (***p < 0.001, ****p < 0.0001, ns not significant). (**b**) *ANOVA* (*p = 1.6 × 10*^*−5*^): N1-M641I, *p = 1.6 × 10*^*−4*^; N1-M641L, *p = 0.59*; N1-M641V, *p = 0.99*; N1-M641T, *p = 0.99*; N2A-V639I, *p = 0.99*. (**c**) *ANOVA* (*p = 1.7 × 10*^*−5*^): N1-M641I, *p = 4.3 × 10*^*−5*^; N1-M641L, *p = 0.38*; N1-M641V, *p = 0.99*; N1-M641T, *p = 0.99*; N2A-V639I, *p = 0.99*.

The missense variant, GluN1-M641I, which is in the vestibule (Figure 1b), reduces Ca^2+^ permeability in the GluN1a/GluN2A receptor^15^. To test if this is a general property of variants in the vestibule, we measured Ca^2+^ permeability using changes in reversal potentials on going from 0 to 10 mM Ca^2+42^ for other variants at GluN1-M641 as well as a variant (isoleucine, I) at the homologous position in GluN2A (V639) (Figure 2). GluN1a(M641I)/GluN2A significantly reduced changes in reversal potentials (ΔE_rev_) (Figure 2b) and correspondingly Ca^2+^ permeability (P_Ca_/P_Na_) (Figure 2c), consistent with previous results^15^. In contrast, other disease variants at GluN1-M641 including a leucine (L), a valine (V), or a threonine (T) do not. Similarly, the variant GluN2A-V639I also does not change Ca^2+^ permeability. Hence, at least in terms of this specific position in the vestibule, changes in Ca^2+^ permeability are not a general property but rather are specific to the isoleucine at GluN1-M641.

### The missense variant GluN1a-M641I induces robust subconductance states

**Figure 3.**
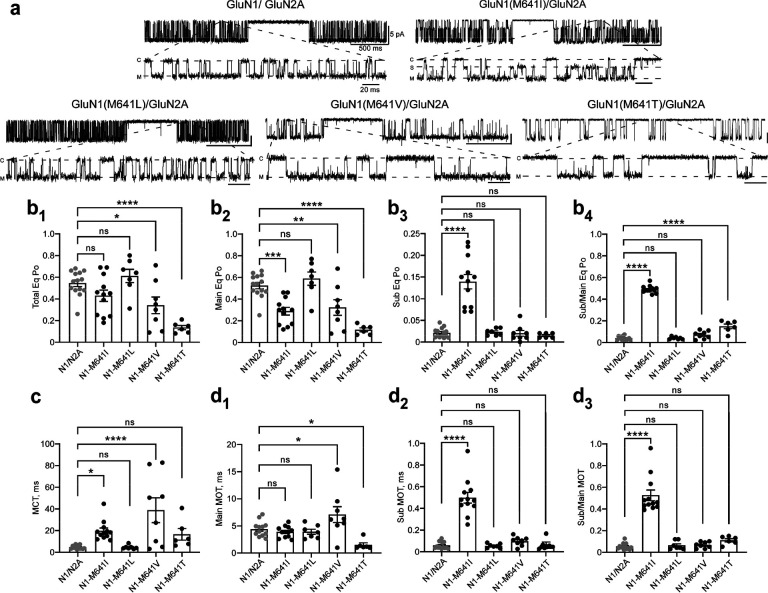
Disease-associated variants at GluN1-M641 expressed with GluN2A alter receptor gating in distinct ways. **(a)** Single-channel currents from human GluN1/GluN2A and GluN1-M641 variants expressed in HEK293T cells. Currents were elicited by 1 mM glutamate and 100 μM glycine at a −100 mV holding potential. For each construct, the upper trace is filtered at 1 kHz and the lower trace shows a higher-resolution segment from the same recording, filtered at 3 kHz. Channel states are denoted as closed (C), subconductance (S), and main (M). Note the prominent subconductance activity in M641I which is minimal in wild type and other variants. **(b)** Bar graphs (mean ± SEM) showing equilibrium open probabilities: total (Total Eq. Po) (**b**_**1**_), main conductance (Main Eq. Po) (**b**_**2**_), subconductance (Sub Eq. Po) (**b**_**3**_), and the ratio of subconductance to main conductance state open probabilities (Sub/Main Eq. Po) (**b**_**4**_). **(c & d)** Bar graphs (mean ± SEM) showing mean closed time (MCT) (**c**) and mean open time for the main (Main MOT) (**d**_**1**_) and sub (Sub MOT) conductance states (**d**_**2**_), and the ratio of open times between sub and main conductance states (Sub/Main MOT) (**d**_**3**_). Statistical comparisons were performed using one-way ANOVA with post hoc Dunnett tests versus wild-type (*p < 0.05, **p < 0.01, ***p < 0.005, ****p < 0.001, ns not significant). (**b**_**1**_) *ANOVA* (*p = 7.5 × 10*^*−6*^): N1-M641I, *p = 0.22*; N1-M641L, *p = 0.8*; N1-M641V, *p = 0.02*; N1-M641T, *p = 1.3 × 10*^*−5*^. (**b**_**2**_) *ANOVA* (*p = 7.1 × 10*^*−8*^): N1-M641I, *p = 2.6 × 10*^*−4*^; N1-M641L, *p = 0.73*; N1-M641V, *p = 6.3 × 10*^*−3*^; N1-M641T, *p = 8.8 × 10*^*−7*^. (**b**_**3**_) *ANOVA* (*p = 2.1 × 10*^*−12*^): N1-M641I, *p = 1.3 × 10*^*−11*^; N1-M641L, *p = 0.99*; N1-M641V, *p = 0.99*; N1-M641T, *p = 0.99*. (**b**_**4**_) *ANOVA* (*p = 1 × 10*^*−15*^): N1-M641I, *p = 1.8 × 10*^*−15*^; N1-M641L, *p = 0.99*; N1-M641V, *p = 0.12*; N1-M641T, *p = 3.9× 10*^*−9*^. (**c**) *ANOVA* (*p = 6.7 × 10*^*−5*^): N1-M641I, *p = 0.048*; N1-M641L, *p = 0.99*; N1-M641V, *p = 2.2 × 10*^*−*^; N1-M641T, *p = 0.33*. (**d**_**1**_) *ANOVA* (*p = 2.1 × 10*^*−4*^): N1-M641I, *p = 0.93*; N1-M641L, *p = 0.97*; N1-M641V, *p = 0.016*; N1-M641T, *p = 0.019*. (**d**_**2**_) *ANOVA* (*p = 1 × 10*^*−15*^): N1-M641I, *p = 1 × 10*^*−15*^; N1-M641L, *p = 0.99*; N1-M641V, *p = 0.77*; N1-M641T, *p =0.97*. (**d**_**3**_) *ANOVA* (*p = 1 × 10*^*−15*^): N1-M641I, *p = 2.8 × 10*^*−15*^; N1-M641L, *p = 0.99*; N1-M641V, *p = 0.99*; N1-M641T, *p = 0.56*. See Supplementary Table 2 and Supplementary Figure 1 for ns and additional parameters.

To begin to define the mechanism underlying the change in Ca^2+^ permeability induced by GluN1-M641I, we recorded single NMDAR channels in on-cell patches using an external solution at pH 7.4, containing EDTA to chelate Zn^2+^, and no added Ca^2+^ (Figure 3, Supplementary Table 2). Under these conditions and comparable to previous publications^23,43^, wild-type GluN1a/GluN2A receptors had a high total equilibrium open probability (Total Eq. Po) of 0.54 ± 0.03 (mean ± SEM, 14 patches recorded) (Total Eq. Po = Sub Eq. Po + Main Eq. Po) (Figure 3b_1_) composed almost exclusively of a main conductance state (Main Eq. Po ~0.52, Figure 3b_2_) (Main amplitude, −7.3 ± 0.2 pA, Supplementary Table 2, Supplementary Figure 1) with a rarely detected subconductance state (Sub Eq. Po ~0.02, Figure 3b_3_) (Sub amplitude, −4.2 ± 0.2 pA). Note that the Sub amplitude in wild-type GluN1a/GluN2A cannot be viewed as rigorous since these events were often intermingled with noise.

The variant GluN1a-M641I when co-expressed with GluN2A showed a slightly reduced Total Eq. Po, about 0.43, which was not significantly different from wild-type (Figure 3b_1_). On the other hand, the Main Eq. Po was significantly reduced (Figure 3b_2_) with the unaffected Total Eq. Po reflecting that GluN1a-M641I spends considerable time in a robust subconductance state with Sub Eq. Po around 0.14 (vs. 0.02 for wild-type) (Figure 3a, 3b_3_). Consequently, the ratio of the Sub to Main Eq. Po was significantly increased (Figure 3b_4_). Finally, the mean open time (MOT) of the Main conductance state was unchanged relative to wild-type (Figure 3d_1_), whereas the Sub MOT was significantly increased (Figures 3d_2_, 3d_3_), strongly supporting a robust subconductance state induced by GluN1-M641I. The Main (-6.6 ± 0.3 pA) amplitude was not different from wild type; the Sub (-2.8 ± 0.1 pA) amplitudes were significantly lower (Supplementary Table 2, Supplementary Figure 1).

To test if enhanced subconductance states are an intrinsic feature of GluN1-M641, we tested single channels of other variants at this position (Figure 3, Supplementary Table 2). GluN1-M641L had no significant effect on Total Eq. Po, while both M641V and M641T significantly reduced it (Figure 3b_1_), with these variants using different mechanisms to change Total Eq. Po: M641V significantly increased mean closed time (MCT) (Figure 3c) whereas M641T significantly decreased the Main MOT (Figure 3d_1_). Nevertheless, and in contrast to the isoleucine variant, the leucine, valine, and threonine variants did not produce a significant subconductance state beyond what was observed in wild type either in terms of Sub Eq. Po (Figure 3b_3_) or Sub MOT (Figure 3d_2_). Hence, the robust subconductance state observed in GluN1-M641I is unique to the isoleucine side chain, not of the M641 position. In short, variants at GluN1a-M641 produce diverse effects on receptor single-channel activity, either producing a robust subconductance state (M641I) or no effect (M641L), or decreasing receptor activity (M641V, M641T).

### GluN1a-M641I when co-expressed with other GluN2 subunits enhances a subconductance level but strongly reduces receptor gating

**Figure 4.**
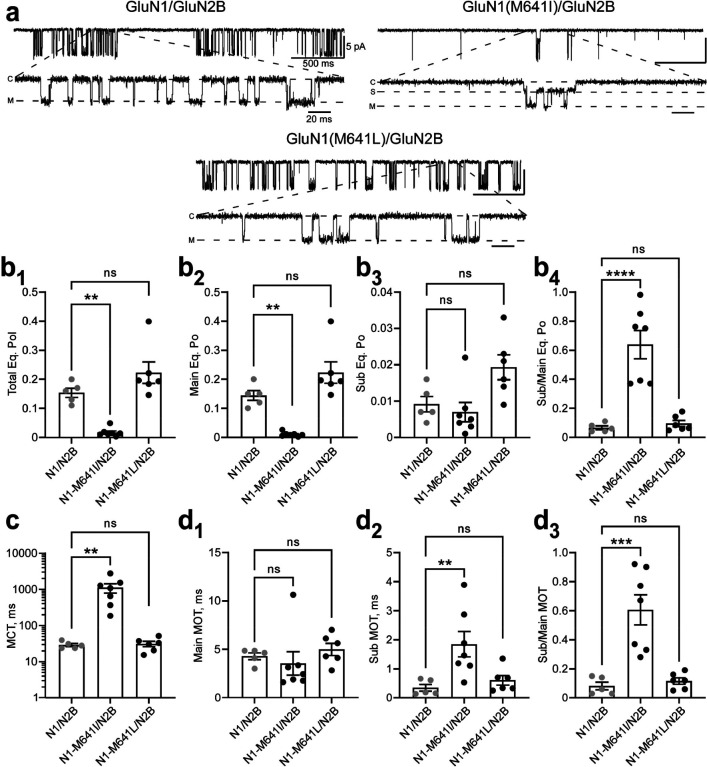
GluN1(M641I) dramatically reduces main conductance open probability of GluN1/GluN2B receptors but retains a prominent subconductance state. (**a**) Single-channel currents from human GluN1/GluN2B, GluN1(M641I)/GluN2B, and GluN1(M641L)/GluN2B. Data recorded and displayed as in Figure 3a. (**b**) Bar graphs (mean ± SEM) showing equilibrium open probabilities. (**c & d)** Bar graphs (mean ± SEM) showing MCT (**c**) and MOT (**d**). Statistical analysis was performed as in Figure 3 (**p < 0.01, ***p < 0.005, ****p < 0.001, ns not significant). (**b**_**1**_) *ANOVA* (*p = 2.6 × 10*^*−5*^): N1-M641I, *p = 1.4 × 10*^*−3*^; N1-M641L, *p = 0.1*. (**b**_**2**_) *ANOVA* (*p = 1.8 × 10*^*−5*^): N1-M641I, *p = 1.7 × 10*^*−3*^; N1-M641L, *p = 0.057*. (**b**_**3**_) *ANOVA* (*p = 0.016*): N1-M641I, *p = 0.81*; N1-M641L, *p = 0.053*. (**b**_**4**_) *ANOVA* (*p = 2 × 10*^*−5*^): N1-M641I, *p = 5.5 × 10*^*−5*^; N1-M641L, *p = 0.94*. (**C**) *ANOVA* (*p = 3.5 × 10*^*−3*^): N1-M641I, *p = 7.4 × 10*^*−3*^; N1-M641L, *p = 0.99*. (**d**_**1**_) *ANOVA* (*p = 0.83*): N1-M641I, *p = 0.79*; N1-M641L, *p = 0.82*. (**d**_**2**_) *ANOVA* (*p = 9.0 × 10*^*−3*^): N1-M641I, *p = 9.6 × 10*^*−3*^; N1-M641L, *p = 0.8*. (D_**3**_) *ANOVA* (*p = 1.4 × 10*^*−4*^): N1-M641I, *p = 3.1 × 10*^*−4*^; N1-M641L, *p = 0.93*. See Supplementary Table 3 and Supplementary Figure 2 for ns and additional parameters.

GluN1 is an obligatory subunit and can be associated with different GluN2 subunits^2^. To further define the impact of GluN1a-M641I on receptor function, we co-expressed it with different GluN2 subunits, including GluN2B (Figure 4, Supplementary Table 3), GluN2C, or GluN2D (Supplementary Figure 2, Supplementary Table 3). Comparable to previous publications^44^, wild-type GluN1a/GluN2B shows, relative to GluN2A, a reduced Total Eq. Po of 0.15 ± 0.02, n = 5 (Figure 4b_1_), which is dominated by the Main conductance (Main Eq. Po around 0.14, Figure 4b_2_) with a small contribution from a subconductance state (Sub Eq. Po around 0.009, Figure 4b_3_).

When co-expressed with GluN2B and in contrast to GluN2A, GluN1a-M641I significantly decreased the Total Eq. Po (0.017 vs 0.15 for wild-type) (Figure 4b_1_), reflecting exclusively a dramatic decrease in the Main Eq. Po (Figure 4b_2_), while leaving the Sub Eq. Po unchanged (Figure 4b_3_). Because of the strong Main Eq. Po decrease, the ratio of the Sub/Main Eq. Po was significantly increased (Figure 4b_4_). When co-expressed with GluN2B, GluN1a-M641I dramatically decreases the overall activity of the receptor (due to significantly increased MCT; Figure 4c) but also enhances the subconductance state as illustrated by the significant increase in the Sub MOT (Figure 4d_2_). Finally, like when co-expressed with GluN2A, the variant GluN1-M641L had no effect on single-channel activity when co-expressed with GluN2B (Figure 4), suggesting that the enhancement of the subconductance state in GluN2B is again exclusive to the isoleucine side chain.

Co-expressing GluN1a-M641I with either GluN2C (Supplementary Figure 2a-d) or GluN2D (Supplementary Figure 2e-h) generated a phenotype comparable to what is observed in GluN2B: the Total Eq. Po was significantly decreased for both GluN2 subunits (Supplementary Figure 5b_1_, 5f_1_) with these effects arising due to a decrease in the Main Eq. Po (Supplementary Figure 5b_2_, 5f_2_). Since the Sub Eq. Po was unchanged for both (Supplementary Figure 5b_3_, 5f_3_), the ratio of the Sub/Main Eq. Po was significantly increased (Supplementary Figure 5b_4_, 5f_4_). Supporting an enhanced subconductance state in GluN2C channels, the Sub MOT was increased (Supplementary Figure 5d_2_). However, this did not occur for GluN2D-containing channels (Supplementary Figure 5h_2_).

In summary, when GluN1a-M641I is co-expressed with other GluN2 subunits, either GluN2B, GluN2C, or GluN2D, in contrast to GluN2A, it dramatically reduces receptor activity, which has unique implications for its pathological actions. Nevertheless, GluN1a-M641I still induces a robust subconductance state when co-expressed with these GluN2 subunits.

### Variants at the homologous position to GluN1-M641I in GluN2A and GluN2D strongly enhance gating but do not generate robust subconductance states

**Figure 5.**
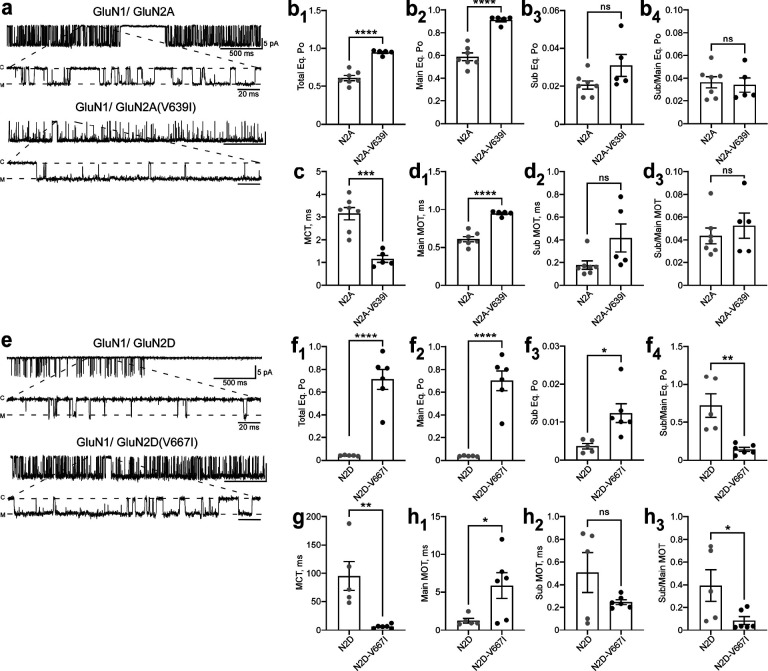
Disease-associated variants GluN2A(V639I) and GluN2D(V667I), homologous positions to GluN1(M641), increase NMDAR open probability. (**a**) Single-channel currents from human GluN1/GluN2A and GluN1/GluN2A(V639I) receptors. (**b**) Bar graphs (mean ± SEM) showing equilibrium open probabilities. (**c & d**) Bar graphs (mean ± SEM) showing MCT (**c**) and MOT (**d**). (**e**) Single-channel currents from human GluN1/GluN2D and GluN1GluN2D(V667I) receptors. (**f**) Bar graphs (mean ± SEM) showing equilibrium open probabilities for constructs in panel E. (**g & h**) Bar graphs (mean ± SEM) showing MCT (**g**) and MOT (**h**). Statistical analysis was performed as in Figure 3 (*p < 0.05, **p < 0.01, ***p < 0.005, ****p < 0.001, ns not significant). (**b**_**1**_) Shapiro–Wilk normality test: N2A, *p = 0.93*; N2A-V639I, *p = 0.03*. Mann–Whitney rank-sum test: N2A-V639I, *p = 2.5 × 10*^*−4*^. (**b**_**2**_) Shapiro–Wilk normality test: N2A, *p = 0.88*; N2A-V639I, *p = 1.2 × 10*^*−3*^. Mann–Whitney rank-sum test: N2A-V639I, *p = 2.5 × 10*^*−4*^. (**b**_**3**_) Shapiro–Wilk normality test: N2A, *p = 0.5*; N2A-V639I, *p = 0.13*. Unpaired two-tailed t-test: N2A-V639I, *p = 0.089*. (**b**_**4**_) Shapiro–Wilk normality test: N2A, *p = 0.43*; N2A-V639I, *p = 0.1*. Unpaired two-tailed t-test: N2A-V639I, *p = 0.77*. (**c**) Shapiro–Wilk normality test: N2A, *p = 0.47*; N2A-V639I, *p = 0.52*. Unpaired two-tailed t-test: N2A-V639I, *p = 2.3 × 10*^*−4*^. (**d**_**1**_) Shapiro–Wilk normality test: N2A, *p = 0.31*; N2A-V639I, *p = 0.85*. Unpaired two-tailed t-test: N2A-V639I, *p = 0.017*. (**d**_**2**_) Shapiro–Wilk normality test: N2A, *p = 3.9 5 × 10*^*−3*^; N2A-V639I, *p = 0.11*. Whitney rank-sum test: N2A-V639I, *p = 0.015*. (**d**_**3**_) Shapiro–Wilk normality test: N2A, *p = 0.080*; N2A-V639I, *p = 0.27*. Unpaired two-tailed t-test: N2A-V639I, *p = 0.48*. (**f**_**1**_) Shapiro–Wilk normality test: N2D, *p = 0.77*; N2D-V667I, *p = 0.53*. Unpaired two-tailed t-test: N2D-V667I, *p = 7.5 × 10*^*−5*^. (**f**_**2**_) Shapiro–Wilk normality test: N2D, *p = 0.82*; N2D-V667I, *p = 0.45*. Unpaired two-tailed t-test: N2D-V667I, *p = 7.3 × 10*^*−5*^. (**f**_**3**_) Shapiro–Wilk normality test: N2D, *p = 0.38*; N2D-V667I, *p = 0.099*. Unpaired two-tailed t-test: N2D-V667I, *p = 0.014*. (**f**_**4**_) Shapiro–Wilk normality test: N2D, *p = 0.10*; N2D-V667I, *p = 0.8*. Unpaired two-tailed t-test: N2D-V667I, *p = 2.8 × 10*^*−3*^. (**G**) Shapiro–Wilk normality test: N2D, *p = 0.23*; N2D-V667I, *p = 0.28*. Unpaired two-tailed t-test: N2D-V667I, *p = 3.9 × 10*^*−3*^. (**h**_**1**_) Shapiro–Wilk normality test: N2D, *p = 0.019*; N2D-V667I, *p = 0.48*. Whitney rank-sum test: N2A-V639I, *p = 0.039*. (**h**_**2**_) Shapiro–Wilk normality test: N2D, *p = 0.79*; N2D-V667I, *p = 0.93*. Unpaired two-tailed t-test: N2D-V667I, *p = 0.14*. (**h**_**3**_) Shapiro–Wilk normality test: N2D, *p = 0.21*; N2D-V667I, *p = 8.7 × 10*^*−3*^. Whitney rank-sum test: N2A-V639I, *p = 0.045*. See Supplementary Table 4 for ns and additional parameters.

At both GluN2A-V639 and GluN2D-V667, which are the homologous positions to GluN1-M641, isoleucine variants have been identified (Supplementary Table 1). To further test the significance of isoleucine positioned in the vestibule, we recorded single-channel activity of GluN2A-V639I and GluN2D-V667I (Figure 5, Supplementary Table 4). In contrast to variants at GluN1-M641, which either did not affect (I, L) or decreased (V, T) Total Eq. Po (Figure 3), the isoleucine variants in GluN2A (Figure 5b_1_) and GluN2D (Figure 5f_1_) significantly enhanced gating activity, most notably in GluN2D (an approximate 17-fold increase). On the other hand, neither GluN2 variant enhanced the subconductance state: GluN2A-V639I had no effect on Sub Eq. Po (Figure 5b_3_), the Sub/Main Eq. Po ratio (Figure 5b_4_), or Sub MOT (Figure 5d_2_); GluN2D-V677I did significantly increase Sub Eq. Po (Figure 5f_3_) but this reflects the enhanced overall activity of this variant since the Sub/Main Eq. Po ratio was actually significantly decreased (Figure 5f_4_). In addition, GluN2D-V667I had no significant effect on Sub MOT (Figure 5h_2_). Hence, GluN2A-V639I and GluN2D-V667I did not induce any notable subconductance state, suggesting that it is the isoleucine specifically at GluN1-M641 that generates this phenotype.

### Molecular dynamics simulations identify interacting partners of GluN1-M641I

**Figure 6.**
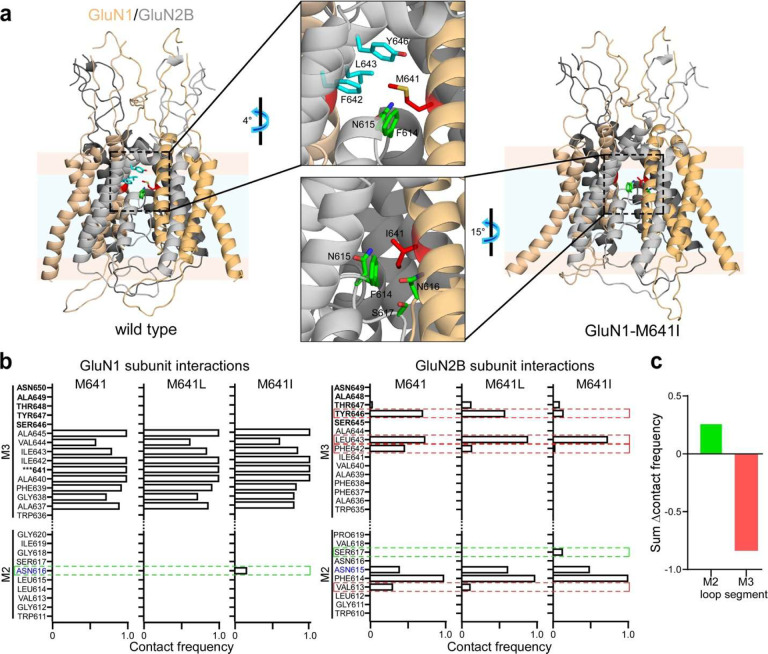
MD simulations of NMDARs and disease-associated variants in the vestibule. **(a)** Representative snapshots from wild-type GluN1/GluN2B (*left*) and GluN1-M641I (*right*) simulations. GluN1 and GluN2B subunits are shown in orange and gray, respectively. GluN1 residue 641 is highlighted in red; several residues within 3.5 Å of residue 641 are shown in cyan (M3) or green (M2). **(b)** Contact frequencies of methionine, leucine, or isoleucine at GluN1–641 with surrounding residues. Colored boxes indicate a reduction (red, < 0.05) or increase (green, > 0.05) in contact frequency when isoleucine is compared to methionine and leucine. The first five resiodues in SYTANLAAF are in bold; N-site asparagines are in blue. **(c)** Sum of the changes in contact frequency for M641I with either the M2 loop or the M3 segment, with the mean of wild type and M641L as reference. Green and red indicate gain and loss of interactions, respectively.

To define the molecular basis of the changes in P_Ca_ and induction of subconductance states in GluN1-M641I, we carried out molecular dynamics (MD) simulations (see Methods) using rat GluN1/GluN2B based on an open state structure, 9ARE^45^ (Figure 6). We carried out simulations for wild type (GluN1-M641), the leucine variant (M641L), which has no effect on P_Ca_ (Figure 2c) nor single channel activity (Figures 3 & 4), and the isoleucine variant (M641I), which reduces P_Ca_ (Figure 2c) and induces subconductance states (Figures 3, 4). We then calculated the contact frequencies of the residue at the GluN1–641 position with other residues. In general, the quantitative results for M and L were similar. Therefore, we used M and L as references to highlight the changes with I.

Overall, there was a strong trend for the isoleucine to have reduced interactions with the M3 segment and increased interactions with the M2 loop, notably the asparagines and a serine around the selectivity filter (Figures 6a, 6b). These differences from methionine and leucine can be attributed to the β-branching of the isoleucine side chain, which forces it to project downward (toward the M2 loop) instead of upward as in the case of methionine and leucine (Figure 6a). In the two GluN1 subunits, whereas methionine and leucine essentially had no contact with the N-site asparagine, isoleucine had a contact frequency of 0.16 (Figure 6b, *left* panel). In the two GluN2B subunits, the contact frequency of isoleucine with the N-site asparagine was 0.49, higher than the counterpart, 0.39, of the methionine (though not of the leucine); contact was also formed with a proximal serine by isoleucine but not by methionine or leucine (Figure 6b, *right* panel). When contact frequencies were summed over the M3 segment or the M2 loop and isoleucine was compared with the average of methionine and leucine, there was a decrease of 0.84 (GluN2B subunits only) in contact frequency with M3 and an increase of 0.27 in contact frequency with M2 (both GluN1 and GluN2B subunits) (Figure 6c).

### Variants at the GluN1 and GluN2A N-site asparagines generate subconductance states

**Figure 7.**
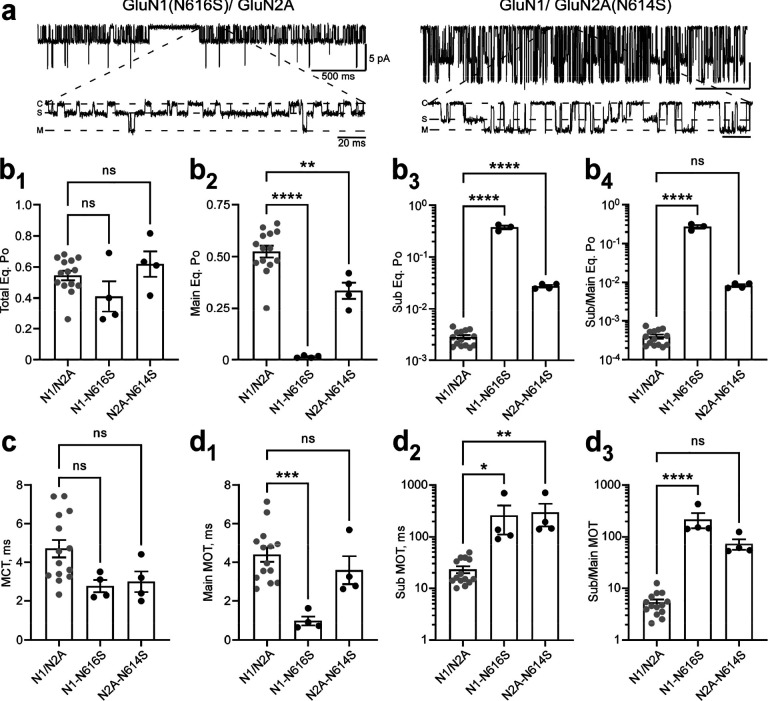
Disease-associated variants (N-to-S) at the GluN1 and GluN2A N-sites induce subconductance states. **(a)** Single-channel currents from human variants GluN1(N616S)/GluN2A and GluN1/GluN2A(N614S). **(b)** Bar graphs (mean ± SEM) showing equilibrium open probabilities for constructs in panel A. **(c & d)** Bar graphs (mean ± SEM) showing MCT (**c**) and MOT (**d**). Statistical analysis was performed as in Figure 3 (*p < 0.05, **p < 0.01, ***p < 0.005, ****p < 0.001, ns not significant). (**b**_**1**_) *ANOVA* (*p = 0.12*): N1-N616S, *p = 0.19*; N2A-N614S, *p = 0.58*. (**b**_**2**_) *ANOVA* (*p = 4.7 × 10*^*−8*^): N1-N616S, *p = 2.2 × 10*^*−8*^; N2A-N614S, *p = 4.3 × 10*^*−3*^. (**b**_**3**_) *ANOVA* (*p = 1.8 × 10*^*−7*^): N1-N616S, *p = 3.8 × 10*^*−7*^; N2A-N614S, *p = 4.8 × 10*^*−5*^. (**b**_**4**_) *ANOVA* (*p = 7 × 10*^*−15*^): N1-N616S, *p = 7 × 10*^*−14*^; N2A-N614S, *p = 0.65*. (**C**) *ANOVA* (*p = 0.043*): N1-N616S, *p = 0.068*; N2A-N614S, *p = 0.11*. (**d**_**1**_) *ANOVA* (*p = 7 × 10*^*−4*^): N1-N616S, *p = 3.3 × 10*^*−4*^; N2A-N614S, *p = 0.48*. (**d**_**2**_) *ANOVA* (*p = 1.4 × 10*^*−6*^): N1-N616S, *p = 0.016*; N2A-N614S, *p = 1.7 × 10*^*−3*^. (**d**_**3**_) *ANOVA* (*p = 1.5 × 10*^*−5*^): N1-N616S, *p = 6.6 × 10*^*−6*^; N2A-N614S, *p = 0.1*. See Supplementary Table 5 and Supplementary Figure 5 for ns and additional parameters.

The MD simulations indicate that the isoleucine variant in contrast to the native methionine and the variant leucine tends to interact less with the M3 segment and more with the M2 loop. Hence, we speculated that the change in P_Ca_ and induction of subconductance states most likely arise from a gain of interaction. Previous studies have indicated that mutations at the N-site can induce subconductance states^46^. We therefore identified variants at the GluN1 and GluN2A N-site asparagines, in both instances an N-to-S, from the literature (Supplemental Table 1) as well as a dataset generated from ClinVAR^47^. Initially, we recorded single-channel activity of these variants (Figure 7, Supplementary Table 5 and Supplementary Figure 5).

Both GluN1-N616S and GluN2A-N614S produced strong subconductance states, with GluN1-N616S spending most of its time in a subconductance level (Figure 7a). Neither variant significantly affected the Total Eq. Po (Figure 7b_1_) or MCT (Figure 7c). Both strongly attenuated the Main Eq. Po (Figure 7b_2_) with GluN1-N616S also significantly reducing the Main MOT (Figure 7d_1_). On the other hand, both significantly enhanced the Sub Eq. Po (Figure 7b_3_) and correspondingly the Sub/Main Eq. Po (Figure 7b_4_) as well as the Sub MOT (Figure 7d_2_). Hence, both GluN1-N616S and GluN2A-N614S strongly enhance subconductance states with GluN1-N616S spending nearly 97% of its open time in the subconductance level.

### Subconductance levels are largely impermeable to Ca^2+^

**Figure 8.**
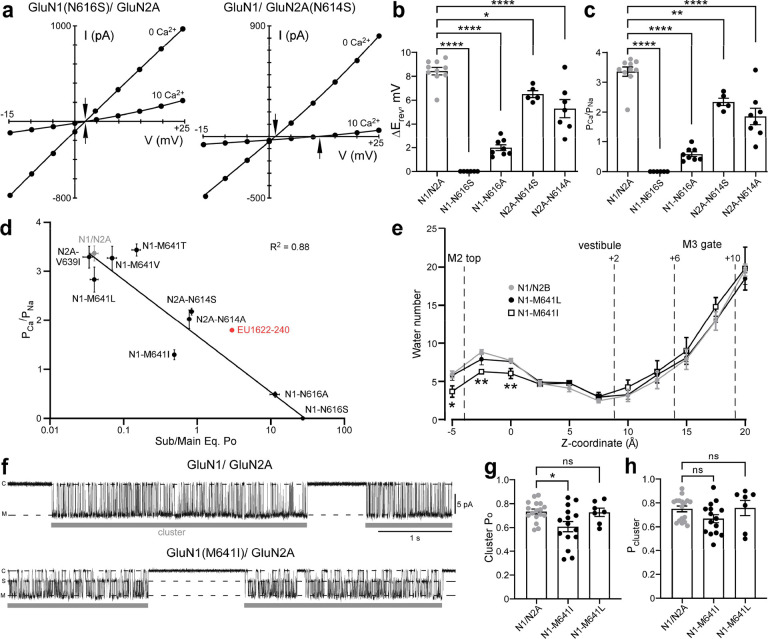
N-site variants and mutations alter P_Ca_. **(a)** NMDAR current-voltage (IV) relationships for disease-associated variants GluN1(N616S)/GluN2A and GluN1/GluN2A(N614S). Data analyzed and displayed as in Figure 2a. **(b & c)** Bar graphs (mean ± SEM) showing changes E_rev_ (ΔE_rev_) (**b**) and relative Ca^2+^ permeability (P_Ca_/P_Na_) (**c**) for N-site variants (N-to-S) and mutations (N-to-A). Number of recordings, from left to right: 10, 6, 7, 5, 8. **(d)** Correlation between P_Ca_/P_Na_ and the ratio of the Sub/Main Eq. Po for variants and mutations measured in the present study. EU1622–240 is a compound that induces subconductance levels and reduces P_Ca_^48^. **(e)** Distribution of waters in the pore. WT (gray circles) and GluN-M641L (black squares), which do not induce robust subconductance states, versus GluN1-M641I (open boxes), which does. **(f)** Example records showing cluster analysis of GluN1/GluN2A and GluN1(M641I)/GluN2A. Gray bars indicate defined clusters (see Methods). **(g & h)** Bar graphs (mean ± SEM) showing Cluster P_o_ (**g**) and P_cluster_ (**h**). Previous work has shown that Cluster P_o_ is an index of the M2 gate whereas P_cluster_ the M3 gate^33^. Statistical analysis was performed as in Figure 2 (*p < 0.05, **p < 0.01, ****p < 0.001, ns not significant). (**b**) *ANOVA* (*p = 4 × 10*^*−15*^): N1-N616S, *p = 6 × 10*^*−15*^; N1-N616A, *p = 8.3 × 10*^*−13*^; N2A-N614S, *p = 0.013*; N2A-N614A, *p = 1 × 10*^*−5*^. (**c**) *ANOVA* (*p = 6 × 10*^*−15*^): N1-N616S, *p = 1.5 × 10*^*−14*^; N1-N616A, *p = 2.6 × 10*^*−13*^; N2A-N614S, *p = 1.5 × 10*^*−3*^; N2A-N614A, *p = 4.7 × 10*^*−7*^. (**g**) *ANOVA* (*p = 0.015*): N1-M641I, *p = 0.012*; N1- M641L, *p = 0.99*. (**h**) *ANOVA* (*p = 0.12*): N1-M641I, *p = 0.15*; N1- M641L, *p = 0.99*. See Supplementary Table 6 for ns and additional parameters.

The isoleucine variant at GluN1-M641, compared to the wild-type methionine and the variant leucine (which has no effect on receptor function), interacts more with elements of the M2 loop including the N-sites (Figure 6). Given that the N-to-S variants also induce subconductance levels, like the M641 isoleucine, we speculate that the action of GluN1-M641I arises from its interactions with the M2 loop. To begin to test this idea, we measured P_Ca_ for the N-to-S variants (Figure 8a–c). To expand the pool for the N-sites, we also introduced a mutation, an alanine (A), at the GluN1 and GluN2A N-sites (GluN1-N616A and GluN2A-N614A). Note that these N-to-A mutations are not disease-associated variants. These N-to-A mutations induced single-channel phenotypes comparable to those for the respective N-to-S variants (Supplementary Figure 6), though there were quantitative differences. For example, GluN1-N616S spent over 97% of its open time in the subconductance state whereas GluN1-N616A spent only 90% of its time in the subconductance state.

We again used changes in reversal potentials on going from 0 to 10 mM Ca^2+^, as in Figure 2, to assay P_Ca_ of the N-site variants and mutations (Figure 8a–c). All N-site variants and mutations strongly attenuated ΔE_rev_ (Figure 8b) and P_Ca_/P_Na_ (Figure 8c), with GluN1-N616S reducing ΔE_rev_ so strongly that we could not reliably detect any Ca^2+^ permeability under the present conditions.

Given the strong effect of N-site variants and mutations in inducing subconductance states (Figure 7, Supplementary Figure 6) and reducing P_Ca_ (Figure 8c), we plotted P_Ca_ against the ratio of the Sub/Main Eq. Po (Figure 8d). Notably, there was a strong negative correlation between P_Ca_ and the relative occupancy of the subconductance state. Hence, subconductance state appear largely impermeable to Ca^2+^.

To begin to address mechanistically how the isoleucine replacement might alter Ca^2+^ permeability and induce subconductance levels, we looked at the distribution of waters in the pore in MD simulations of GluN1-M641, M641L, and M641I (Figure 8e). The waters in GluN1-M641 and M641L were comparable. In contrast, the waters in M641I showed a strong reduction around the M2 loop (Figure 8e). Hence, with the increased interaction with the M2 loop, waters were displaced from this region, which we interpret as underlying the change in P_Ca_ and inducing subconductance states.

As an additional test of the mechanism of action of GluN1-M641I, we characterized single channel activity using cluster analysis (Figure 8f–h, Supplementary Table 6). Previously, we found that the probability of being in a cluster (P_cluster_) was an index of the M3 gate, whereas the open probability during a cluster (Cluster P_o_) was an index of the M2 gate^33^. We therefore used cluster analysis on GluN1/GluN2A, GluN1(M641I)/GluN2A, and GluN1(M641L)/GluN2A. Notably, consistent with the idea that Ile641 interacts with the M2 gate, we saw a significant reduction in Cluster P_o_ (Figure 8g) but no significant change in P_custer_ (Figure 8h).

## DISCUSSION

Of the tested variants in the vestibule, GluN1-M641I is unique: it reduces P_Ca_ (Figure 2) and induces a robust subconductance level (Figure 3, Supplementary Figure 1). Based on MD simulations and, in comparison to the wild-type methionine or the variant leucine which has no effect on P_Ca_ or single channel activity, we find that the isoleucine side chain at GluN1-M641 tends to lose interactions with the M3 segment and gain interactions with the M2 loop (Figure 6), display reduced waters around the M2 loop (Figure 8e), and show decreased Cluster P_o_ (Figure 8g), an index of the M2 gate^33^. We also find that variants or mutations at the N-site induce subconductance levels and reduce P_Ca_ (Figures 7 and 8, Supplementary Figures 5 and 6). We therefore conclude that the isoleucine residue at GluN1-M641 most likely induces its unique actions by interacting and altering the M2 loop. We also conclude that subconductance states are largely impermeable to Ca^2+^ (Figure 8d).

Elements of the M2 loop, notably the N-site and N+1 site asparagines (Figure 1), have previously been shown to have strong effects on Ca^2+^ permeability as well as Mg^2+^ block^49–51^. Further supporting the interaction of GluN1-M641I with the M2 loop is that it strongly attenuates Mg^2+^ block^15,41^, whereas other variants including GluN1-M641L and M641V as well as GluN2A-V639I have either no effect (GluN1-M641L) or very limited effects (GluN1-M641V, GluN2A-V639I) on Mg^2+^ block^41^. In addition, previous work has shown that subconductance states and P_Ca_ and Mg^2+^ sensitivity are linked^52^. Mutating GluN2A-Ser632 to Leu resulted in induced subconductance states in NMDARs as well as reduced Ca^2+^ permeability and Mg^2+^ sensitivity. Oppositely, receptors with the reverse mutation at the homologous site in GluN2D (Leu657) displayed decreased subconductance states and increased Ca^2+^ permeability.

NMDAR have diverse functional properties that contribute to their distinct role in brain development and synaptic dynamics^2^ and to properly characterize whether a variant is GoF or LoF requires assaying a wide range of these parameters. Our experiments characterized only a small number of functional properties of the vestibule variants, including Ca^2+^ permeability (Figure 2) and single channel activity (Figures 3–5, Supplementary Figure 2). In terms of receptor gating, GluN1-M641 variants either had no effect on Total Eq. Po (M641I, M641L) or generated a LoF (M641V, M641T) (Figure 3b_1_). In contrast, variants at the homologous position in GluN2A-V639I or GluN2D-V667I had strong GoF phenotypes (Figures 5b_1_, f_1_) with GluN2D-V667I increasing receptor opening nearly 17-fold. The molecular basis for this difference between GluN1 and GluN2A is unknown though in this region GluN2 tends to have stronger effects on receptor function than GluN1^35^.

Since GluN1 is obligatory, we expressed GluN1-M641I with different GluN2 subunits either GluN2A (Figure 3), GluN2B (Figure 4) or GluN2C or GluN2D (Supplementary Figure 2). In all instances, GluN1-M641I induced robust subconductance states, though less notable in GluN2D. However, when co-expressed with GluN2A, there was only a small albeit not significant effect on Total Eq. Po (Figure 3b_1_). In contrast, when co-expressed with either GluN2B (9-fold reduction), GluN2C (5.2-fold reduction), or GluN2D (4.7-fold reduction), there was a strong reduction in receptor activity, which will have strong implications for GluN1-M641I. Overall, because of a reduction in Mg^2+^ block, GluN1-M641I, when co-expressed with GluN2A, is a GoF^15,40,41,53^. Nevertheless, the LoF in intrinsic Ca^2+^ permeability alters the overall profile^15^. In terms of GluN2B, GluN2C, and GluN2D, GluN1-M641I is most likely a LoF even if Mg^2+^ block is attenuated. In addition, we were unable to measure the impact of GluN1-M641I in these subunits on P_Ca_ because of extremely low whole-cell current amplitudes. Nevertheless, we surmise that they will have extremely low Ca^2+^ permeability, further adding to their LoF phenotype.

Previously it has been proposed that subconductance states arise from alterations in the M3 gate^31^. For our experiments, we focused on variants either in the vestibule or the M2 loop that generated subconductance states. For both, we assume that subconductance states arise from variations in the topology of the M2 loop. Notably, the compound used to demonstrate subconductance states (EU1622–240), also interacts with the M2 loop^31^. Remarkably, similar to GluN1-M641 and N-site variants and mutations, EU1622–240 reduces NMDARs Ca^2^^+^ permeability and Mg^2^^+^ sensitivity^48^. Another positive allosteric modulator that induces subconductance states, EU1644–14, has also been shown to attenuate Ca^2^^+^ permeability and Mg^2^^+^ block^54^. Nevertheless, variants that induce subconductance states in more extracellular regions, if they can be identified, will need to be tested to refine our understanding whether subconductance states occur at the M2 loop, the M3 gate, or both.

Our experiments highlight a unique phenotype for GluN1-M641I. Outcomes with this variant as well as others in the vestibule and the M2 loop indicate a strong negative correlation between P_Ca_ and the extent of subconductance states (Figure 8d), which we interpret to reflect that subconductance states are largely impermeable to Ca^2+^. Variants can alter P_Ca_ without inducing subconductance states^55^. Still, the implication is that variants that induce subconductance states will have a reduced Ca^2+^ influx, which will have a profound impact on clinical phenotypes.

## METHODS

### Molecular biology

All manipulations were made in human GluN1 (hGluN1; Q05586), GluN2A (hGluN2A; Q12879), GluN2B (hGluN2B; Q13224), GluN2C (hGluN2C; Q14957), and GluN2D (hGluN2C; O15399) subunits, which were generously provided by Drs. Stephen Traynelis and Hongjie Yuan (Emory University). In all instances, numbering included the signal peptide (GluN1, 18 residues; GluN2A, 19 residues GluN2B, 26 residues; hGluN2C, 19 residues; GluN2D; 26 residues). Mutations were generated with Q5 site-directed mutagenesis kit (New England Biolabs) with NEB 5-alpha competent cells. Plasmid DNA was isolated using QIAprep Spin Miniprep Kit (Qiagen) and sequenced to verify the presence of the variant.

### Cell Culture and Transfection

For details on cell culture and transfection^56^. Briefly, human embryonic kidney 293T (HEK 293T) were grown in Dulbecco’s modified Eagle’s medium (DMEM), supplemented with 10% fetal bovine serum (FBS) at 37°C in 5% CO_2_. cDNA constructs in a pCI-neo vector were co-transfected into HEK 293T cells along with a separate pEGFP-Cl vector at a ratio of 4:2:1 (N1/N2/EGFP for NMDARs) using X-tremeGene HP (Roche). To improve cell survivability, HEK 293T cells were bathed in a media containing the NMDAR competitive antagonist APV (100 μM) and Mg^2+^ (1 mM). All experiments were performed 18–36 hours post-transfection. Successfully transfected cells were identified by GFP fluorescence.

### Single-channel recording

On-cell single-channel recordings were collected at room temperature (20–23°C) at a −100 mV holding potential. Currents were recorded using a patch clamp amplifier (Axopatch 200B; Molecular Devices), filtered at 10 kHz (four-pole Bessel filter), and digitized at 40 kHz (ITC-16 or ITC-18 interfaced with PatchMaster). The bath solution contained (in mM): 150 NaCl, 10 HEPES, 0.05 EDTA, 1 MgCl_2_. The pipette solution contained (in mM): 150 NaCl, 10 HEPES, 0.05 EDTA, 1 glutamate, and 0.1 glycine, pH 7.4 (NaOH). EDTA was used to minimize the inhibitory effect of Zn^2+ 57^. Recording pipettes were pulled from thick-wall borosilicate capillary glass (Sutter Instruments) and fire-polished to final pipette resistances ranging from 5–12 MΩ when measured in the bath solution (with an applied positive pipette pressure of approximately 100 mbar). Since stray pipette capacitance may raise background noise and thereby reduce the signal-to-noise ratio^58^, we often coated recording pipettes with Sylgard No. 184 (Dow Corning) to thicken their walls and the depth of bath solution in contact with the pipette wall was minimized. Experiments ran for approximately 3–60 min to ensure a significant number of events for analysis.

### Single channel analysis

Analysis of single-channel records was comparable to Talukder and Wollmuth^59^. Data were exported from PatchMaster into QuB for processing. Artifacts and brief segments with excessive noise were removed by aligning them with the adjacent event levels using the “erase” function in QuB. Baseline drift was corrected by resetting the baseline to zero current.

Processed traces were idealized using the segmental k-means (SKM) algorithm in QuB with a dead time of 20 μs. Idealization employed a three-state model representing the closed, subconductance, and main conductance states. Amplitude levels for each state were determined from short record segments containing all conductance levels, analyzed using the “grab” function.

Data analysis was performed on recorded patches containing only one active channel. Recordings of GluN2A subunit-containing NMDARs typically exhibited a relatively high equilibrium open probability (Eq. Po) and numerous events, which facilitated the identification of single-channel patches since multiple channels may result in overlapping openings. For the NMDARs with a low Eq. Po (<0.02), such as those containing GluN2C or GluN2D subunits, we performed statistical tests^60^ for verification that only one active channel was present in the patch (Supplementary Figure 4).

Kinetic analysis was performed in QuB using the maximum interval likelihood (MIL) algorithm with a dead time of 20 μs^33,44,57^. Recordings were fitted with fully liganded linear state models containing three closed states (C1-C3), two desensitized states (C4-C5), two to three conductance states(S1-S3), and two to four main open states(M1-M4). Models with progressively increasing numbers of open and closed states were evaluated until the improvement in log-likelihood (LL) was less than 10 LL units per added state. Clusters of channel activity were identified using a critical time (T_crit_) determined by minimizing the false event ratio between the third (C3) and fourth (C4) closed kinetic states. The equilibrium open probability within clusters (cluster P_open_) was then calculated. Additionally, the probability of a channel being in a cluster (P_cluster_) was determined as:

Eq(1) $$P_{\text{cluster}}=\frac{\text{mean}\;\text{cluster}\;\text{length}}{\text{mean}\;\text{cluster}\;\text{length}+\text{mean}\;\text{intercluster}\;\text{length}}$$

### Whole-cell recordings

Whole-cell patch-clamp electrophysiology was used for evaluation of NMDAR Ca^2+^ permeability. Whole-cell recordings were performed on HEK293T cells at room temperature (20–23°C) using an EPC-10 amplifier with Patchmaster software (HEKA Elektronik, Lambrecht, Germany), digitized at 10 kHz and low-pass filtered at 2.8 kHz (−3 dB) using an 8-pole low pass Bessel filter. Patch microelectrodes were filled with an internal solution consisting of 140 mM KCl, 2 mM NaCl, 10 mM HEPES, 4 mM Mg_2_ATP, 0.3 mM GTP, and 1 mM BAPTA (pH 7.3, KOH). The extracellular solution consisted of 140 mM NaCl, 10 mM HEPES, pH 7.4 (NaOH). NMDAR responses were evoked with 100 μM glycine and 1 mM glutamate. Extracellular solutions were applied using a piezo-driven double barrel application system^61^.

Pipettes had resistances of 4–8 MΩ when filled with the internal solution and measured in the standard external solution. We did not use series resistance compensation nor did we correct for junction potentials.

### Ca2+ permeability analysis

To quantify Ca^2+^ permeability, we measured changes in reversal potentials, ΔE_rev_, for glutamate-activated currents on replacing a reference solution (standard ECS without added Ca^2+^) with a test solution (the same solution but with added 10 mM CaCl_2_). Ca^2+^ permeability ratios (P_Ca_/P_Na_) were calculated using the Lewis equation^42^.

Eq(2) $$\Delta E_{\text{rev}}=\frac{\text{RT}}{F}\text{ln}\left(1+\frac{P_{\text{Ca}}^{’}\;4{\left[Ca^{2+}\right]}_{o}}{P_{\text{Na}}{\left[Na^{+}\right]}_{o}}\right)$$

where R is the gas constant, T is temperature, F is the Faraday’s constant, and

Eq(3) $$P_{\text{Ca}}^{’}=\frac{P_{\text{Ca}}}{1+e\left(\frac{E_{\text{rev},\text{Ca}}\;F}{\text{RT}}\right)}$$

#### Molecular modeling of LBD-TMD constructs in the open state

As in previous studies^33,55^, our computational work focused on a construct encompassing the TMD and LBD-TMD linkers in an open state. The linkers were restrained in MD simulations to maintain the open state. Our open-state model started with the recent open structure 9ARE for rat GluN1/N2B^45^. In our construct, GluN1 contained the following residues: Leu541-Pro670 for M1-M3 and Arg794-Lys841 for M4; GluN2B contained the following residues: Met537-Lys669 for M1-M3 and Gly799-Gln845 for M4. Each fragment was capped with N-terminal acetylation and C-terminal amidation. Missing residues were added using the corresponding sequences (Uniport P35439 for N1 and Q00960 for GluN2B) and Modeller 9.22^62^. The extracellular portion of the GluN2B M3 helices was disrupted; consequently, in trial MD simulations, the pore quickly collapsed. We thus remodeled the SYTANLAAFMI segment as a curved helix. The remodeled structure remained open in MD simulations.

Two mutant constructs (M641I and M641L) were prepared by replacing the M641 residues in the GluN1 subunits with either leucine or isoleucine. Using the CHARMM-GUI membrane builder server^63^ (https://www.charmm-gui.org/), each construct was embedded in a POPC lipid bilayer (116 in outer leaflet and 107 inner leaflet) and solvated by 34,117 water molecules. Na^+^ and Cl^−^ ions were added to neutralize and maintain 150 mM NaCl salt concentration. The dimensions of the simulation box were 100 Å × 100 Å × 153 Å.

#### Molecular dynamics simulations of LBD–TMD constructs in the open state

The CHARMM-GUI six-step equilibration protocol^63^ was used to equilibrate each system in NAMD 3.0^64^. After energy minimized for 10,000 conjugate-gradient cycles, the first three equilibration steps were 125 ps each with a time step of 1 fs, while the subsequent three steps were 500, 500, and 1000 ps with a time step of 2 fs. Steps 1 and 2 were at constant NVT, while steps 3–6 were at constant NPT. Harmonic restraints on lipid headgroup atoms and protein backbone heavy atoms were gradually reduced.

An additional 2 ns equilibration was performed at constant NPT, during which only backbone atoms of the linker residues (residues 541–544, 667–670, and 794–797 in GluN1; residues 537–540, 666–669, and 799–802 in GluN2B) were restrained with a force constant of 5.0 kcal mol^−1^ Å^−2^ to maintain the open conformation. Production simulations were then carried out for 500 ns in four independent replicates for each construct, again with restraints applied only to the linker tip residues at the same force constant. Trajectories were saved at 100 ps interval. The final 250 ns of each trajectory were used for analysis.

In all the simulations, the CHARMM36^65^ force field was used for protein and lipid and the TIP3P model^66^ was used for water. Bonds connected to hydrogens were constrained by the SHAKE algorithm^67^. The cutoff distance for nonbonded interactions was 10 Å; long-range electrostatic interactions were treated by the particle mesh Ewald method^68^. The system temperature was controlled at 300 K using the Langevin thermostat. Pressure was maintained at 1 atm using the Langevin piston barostat^69^.

#### MD analysis

Contact frequencies were calculated using CPPTRAJ^70^, with a 3.5 Å cutoff between heavy atoms of residue 641 in GluN1 and other residues. The number of water molecules was calculated using a custom Tcl script. In each slice of 2.5-Å thickness along the pore axis, the number of waters was counted. Pymol (https://pymol.org) was used to render images.

### Statistical analysis

Data analysis was performed using IgorPro, QuB, Excel, and GraphPad. All average values are presented as mean ± SEM. The number of replicates is indicated in a table associated with the figure. Data distribution was evaluated by the Shapiro-Wilk normality test. Parametric statistics (unpaired Studen’s t-test or ordinary one-way ANOVA) was used for data sets with normal distribution whereas nonparametric statistics (Wilcoxon-signed rank test, Mann–Whitney rank-sum test, or Kruskal–Wallis one-way ANOVA on ranks) was used for data sets without normal distribution. At multiple group comparisons, post hoc Dunnet’s (for comparison of a control mean with the other means) or Tukey test (for comparison of every mean with every other mean) was performed when a significant difference had been found. Unless otherwise noted, statistical significance was set at *p* < 0.05.

## Supplementary Material

Supplementary Files

This is a list of supplementary files associated with this preprint. Click to download.

- SupplementalInformationM641.pdf

## Data Availability

Igor and/or Excel files of all raw data is available upon request.
